# Supplementary material for: Protein Adsorption onto Modified Porous Silica by Single and Binary Human Serum Protein Solutions
Source: Int J Mol Sci. 2021 Aug 25;22(17):9164. doi: 10.3390/ijms22179164 (PMC8430731; doi:10.3390/ijms22179164)
Supplement: Supplementary file 1 [file ijms-22-09164-s001.zip › ijms-1335525-supplementary.pdf]

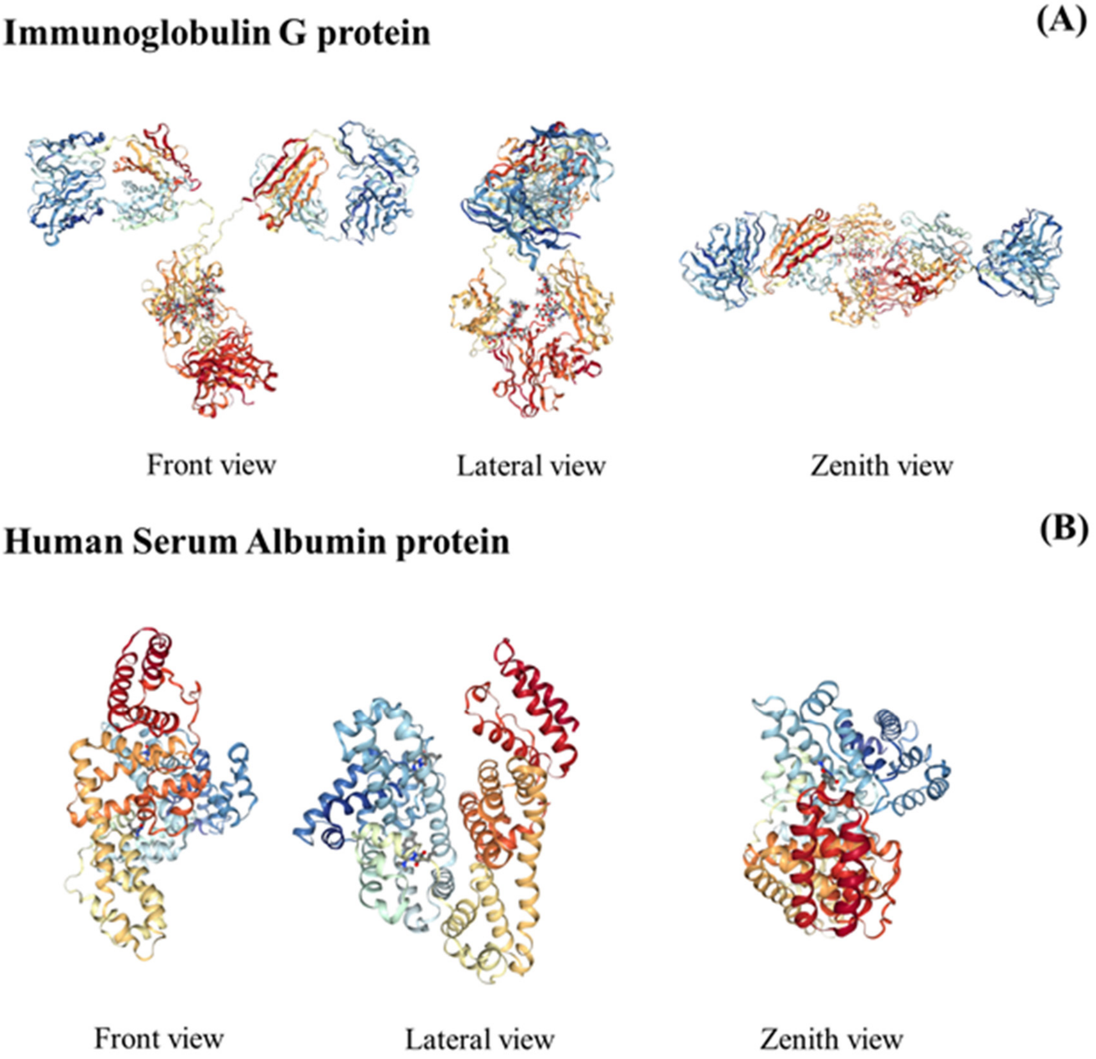

Figure S1. Crystal structure of IgG (A) and HSA (B). Source: Protein Data Bank.

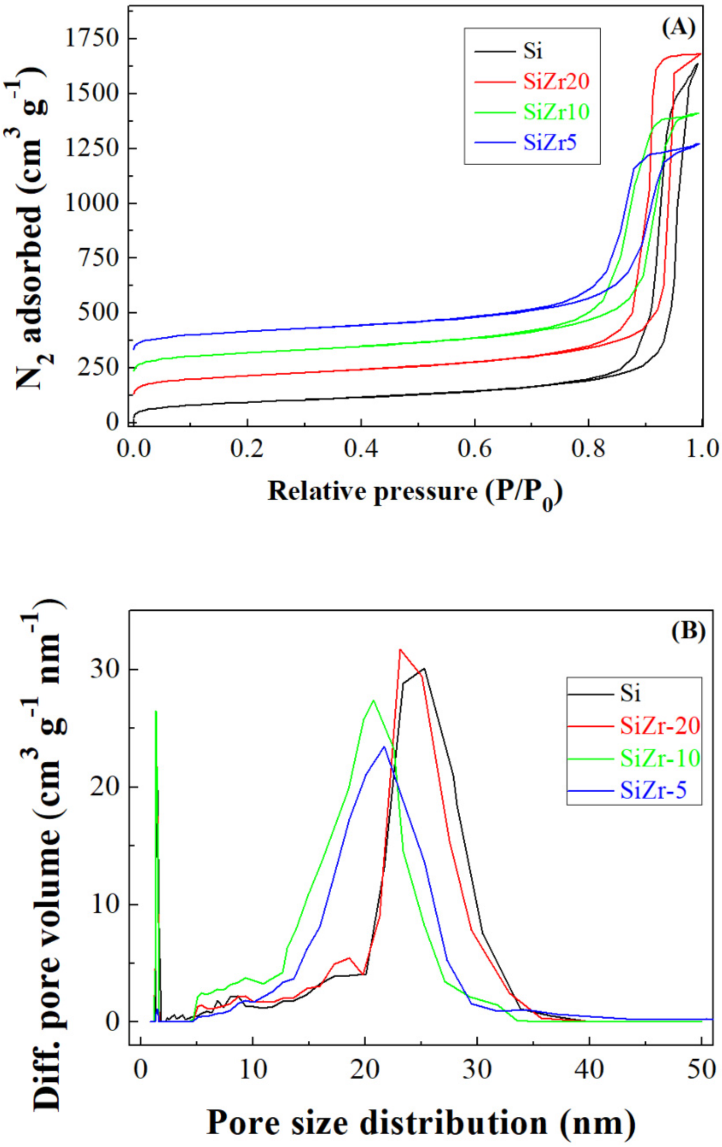

Figure S2. N_2 adsorption desorption isotherms at -196 ºC (A) and pore size distribution estimated from the DFT method (B) of the Si, SiZr20, SiZr10 and SiZr5 samples.

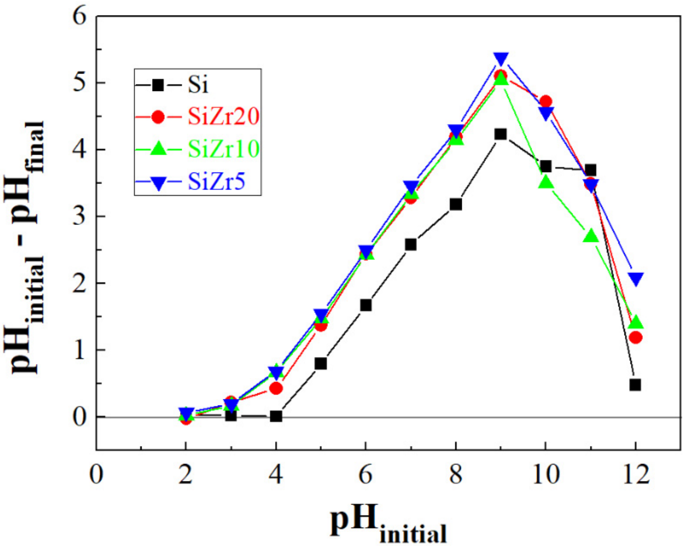

Figure S3. Zero point charge (pH_{ZPC}) of the Si, SiZr20, SiZr10 and SiZr5 samples.

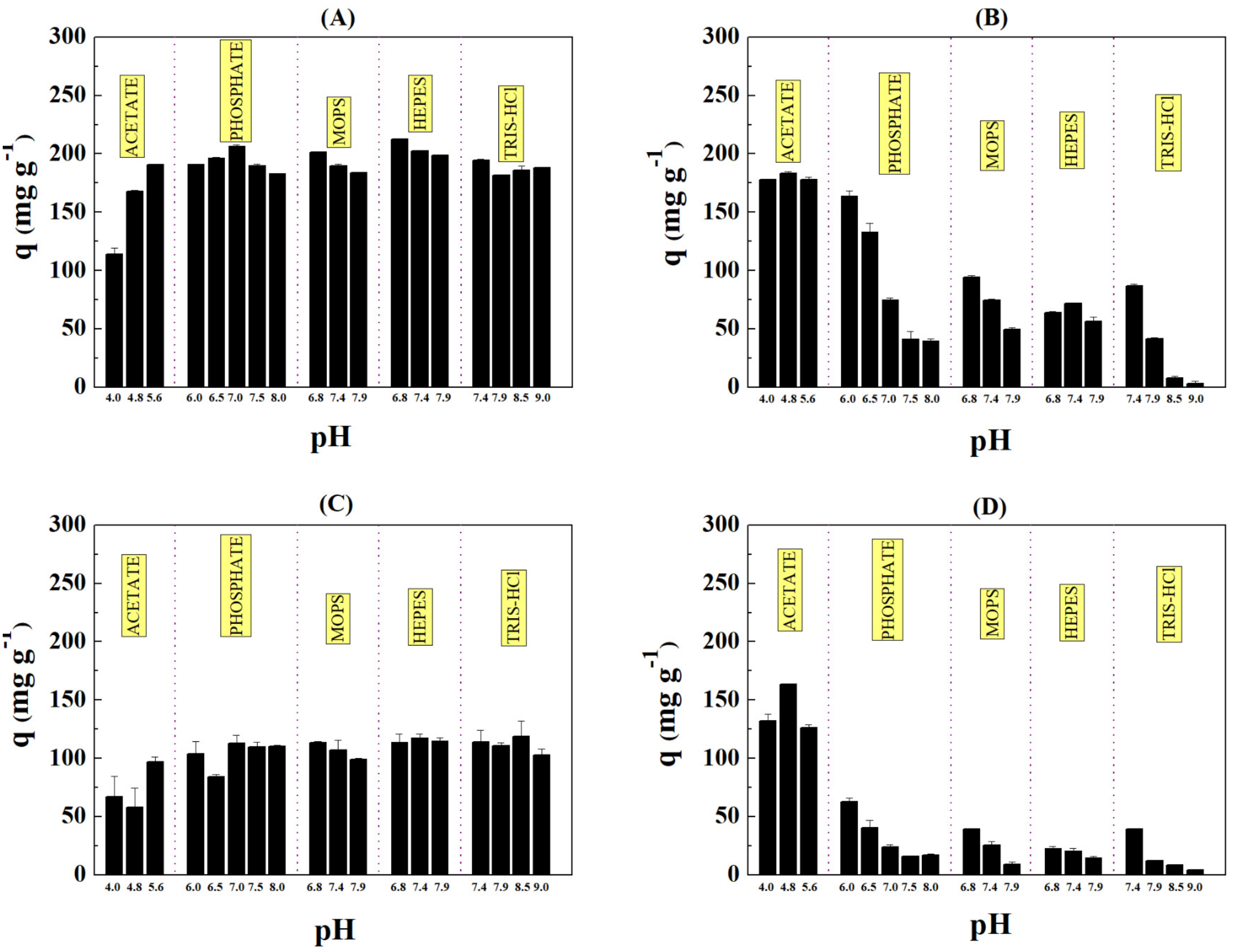

Figure S4. Influence of pH range on the adsorption capacity of IgG and HSA ($C_{initial}$: 1.0 mg mL^{-1}). (A) Si sample with IgG, B) Si sample with HSA, C) SiZr5 with IgG e D) SiZr5 with HSA. pH range of each buffer: acetate (4.0-5.6), phosphate (6.0-8.0), MOPS (6.5-7.9), HEPES (6.8-8.0) and TRIS (7.2-9.0).

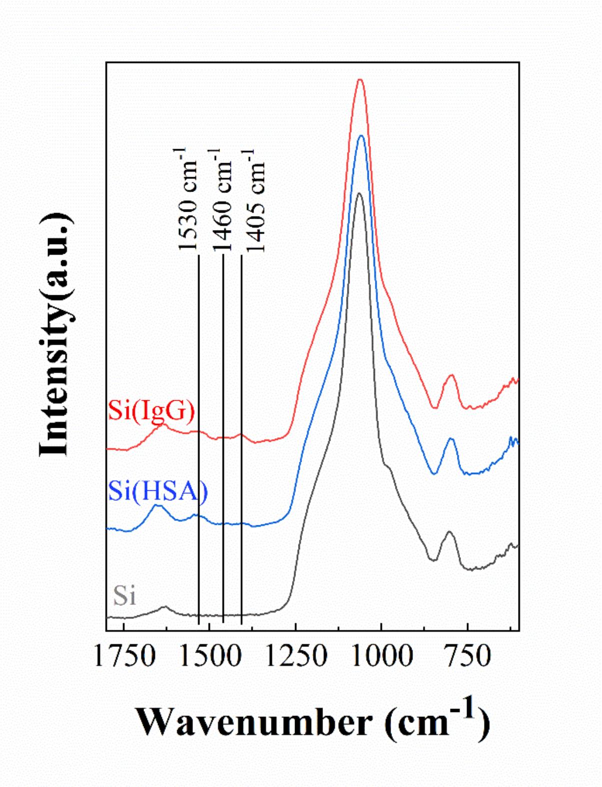

Figure S5. FT-IR spectra of the Si-sample before and after the IgG and HSA adsorption.

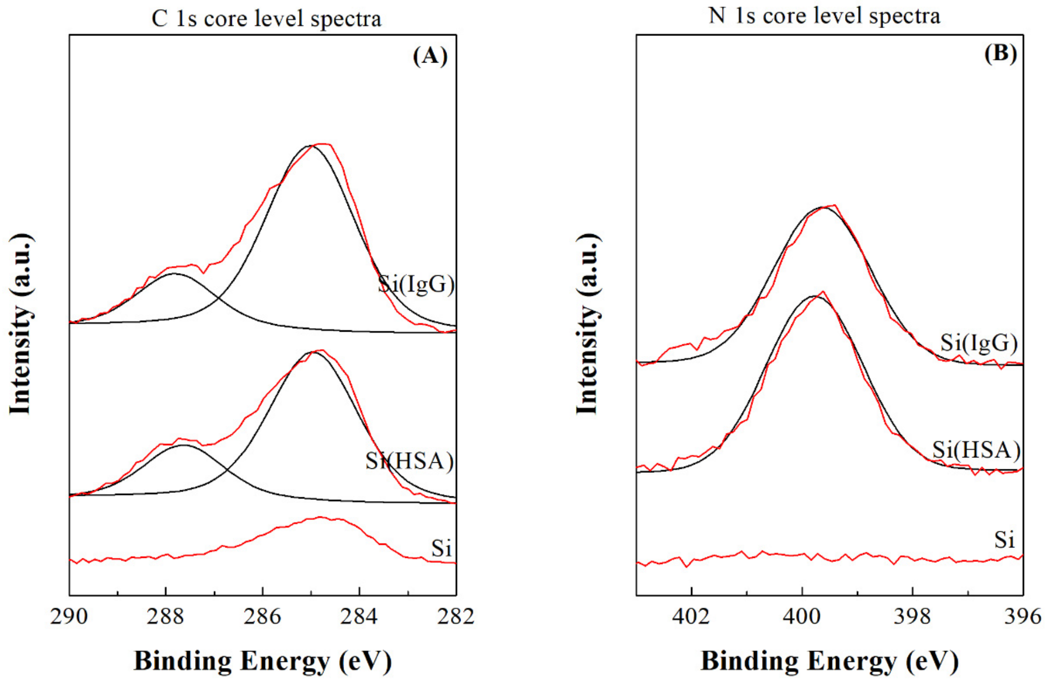

Figure S6. XPS spectra of the Si-sample before and after the IgG and HSA adsorption. C 1 core level spectra (A) and N 1s core level spectra (B).

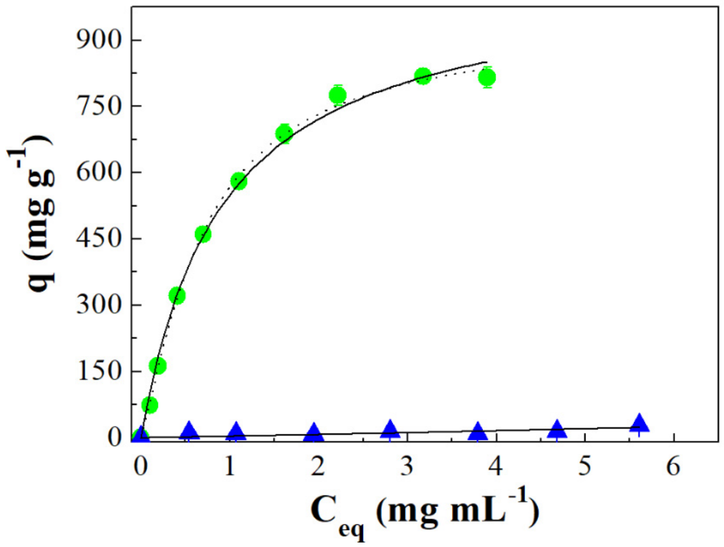

Figure S7. Adsorption isotherm of IgG (●) and HSA (▲) in Si adsorbent (B) using TRIS-HCl at pH 9.0. Adjusted models of Langmuir (dashed black line) and Langmuir-Freundlich (continuous black line).

Table S1. Binary (IgG/HSA) protein concentration diluted in TRIS/HCl 25 mM buffer at pH 9.0 for each case tested.

Binary concentration (mg mL^{-1})	CASE I		CASE II		CASE III	
	IgG (%) 25	HSA (%) 75	IgG (%) 50	HSA (%) 50	IgG (%) 75	HSA (%) 25
	IgG or HSA concentration (mg mL^{-1})					
	IgG	HSA	IgG	HSA	IgG	HSA
0.5	0.25	0.75	0.50	0.50	0.75	0.25
1.0	0.50	1.5	1.0	1.0	1.5	0.50
2.0	1.0	3.0	2.0	2.0	3.0	1.0
4.0	2.0	6.0	4.0	4.0	6.0	2.0
5.0	2.5	7.5	5.0	5.0	7.5	2.5
6.0	3.0	9.0	6.0	6.0	9.0	3.0

Table S2. Langmuir (L) and Langmuir-Freundlich (LF) parameters for the IgG and HSA adsorption in silica and Zr-doped mesoporous silica using acetate buffer at pH 4.8.

**Parameters**	**Si**		**SiZr20**		**SiZr10**		**SiZr5**	
	L	LF	L	LF		L	LF	L
				**IgG (pH=4.8)**				
q_{max} (mg g^{-1})	454.7 ± 36.9	594.9 ± 30.8	215.1 ± 20.5	252.6 ± 89.5	140.4 ± 5.6	192.5 ± 27.1	107.1 ± 4.9	111.7 ± 15.0
k_L (mL mg^{-1})	36.0 ± 12.9	-	1.4 ± 0.51	-	3.9 ± 0.83	-	8.5 ± 2.6	-
k_{LF} (mL mg^{-1})	-	4.6 ± 0.26	-	0.93 ± 0.42	-	0.56 ± 0.23	-	8.2 ± 2.5
R^2	0.92	0.95	0.92	0.99	0.98	0.99	0.96	0.96
N	-	10.1 ± 3.3	-	0.56 ± 0.22	-	0.34 ± 0.18	-	0.80 ± 0.17
				**HSA (pH=4.8)**				
q_{max} (mg g^{-1})	578.7 ± 16.4	607.2 ± 40.3	628.1 ± 34.4	591.9 ± 17.1	423.5 ± 8.4	507.2 ± 40.3	298.1 ± 8.9	355.0 ± 29.0
k_L(mL mg^{-1})	11.6 ± 2.0	-	3.47 ± 1.4	-	2.33 ± 1.1	-	2.9 ± 0.7	-
k_{LF}(mL mg^{-1})	-	18.9 ± 2.1	-	10.6 ± 1.4	-	4.9 ± 1.1	-	3.6 ± 0.9
R^2	0.99	0.99	0.94	0.99	0.99	0.99	0.98	0.99
N	-	0.84 ± 0.15	-	2.0 ± 0.69	-	0.84 ± 0.15	-	0.67 ± 0.17

Table S3. Langmuir (L) and Langmuir-Freundlich (LF) parameters for the IgG and HSA adsorption in silica and Zr-doped mesoporous silica using phosphate buffer at pH 7.0.

**Parameters**	**Si**		**SiZr20**		**SiZr10**		**SiZr5**	
	L	LF	L	LF	L	LF	L	LF
				**IgG (pH=7.0)**				
q_{max} (mg g^{-1})	689.0 ± 20.9	691.4 ± 29.4	311.3 ± 29.4	325.8 ± 7.3	378.9 ± 44.2	386.5 ± 31.5	333.2 ± 36.5	330.2 ± 21.5
k_L(mL mg^{-1})	17.44 ± 2.7	-	7.73 ± 4.4	-	3.96 ± 1.81	-	1.96 ± 0.21	-
k_{LF}(mL mg^{-1})	-	17.2 ± 4.0	-	7.3 ± 3.91	-	4.0 ± 2.21	-	2.3 ± 0.75
R^2	0.98	0.98	0.83	0.88	0.83	0.87	0.89	0.90
N	-	0.98 ± 0.19	-	0.83 ± 0.27	-	0.88 ± 0.35	-	0.83 ± 0.19
				**HSA (pH= 7.0)**				
q_{max} (mg g^{-1})	232.8 ± 8.9	215.1 ± 14.5	648.4 ± 73.3	407.2 ± 50.4	539.1 ± 57.1	428.6 ± 55.0	106.7 ± 18.6	78.4 ± 6.9
k_L (mL mg^{-1})	10.4 ± 2.4	-	16.7 ± 3.7	-	12.0 ± 2.54	-	18.5 ± 8.7	-
k_{LF} (mL mg^{-1})	-	1.18 ± 0.24	-	5.73 ± 0.73	-	10.7 ± 3.1	-	13.1 ± 3.3
R^2	0.99	0.99	0.99	0.98	0.99	0.99	0.91	0.95
N	-	1.17 ± 0.16	-	1.28 ± 0.11	-	1.1 ± 0.15	-	2.2 ± 0.31
